# Stem cells in end-to-side neurorrhaphy. Experimental study in rats^1^

**DOI:** 10.1590/ACB351207

**Published:** 2021-01-20

**Authors:** Geruza Rezende Paiva, Fausto Viterbo, Elenice Deffune, Maria Aparecida Domingues Custódio

**Affiliations:** I Fellow PhD degree, Postgraduate Program in General Basis of Surgery, Universidade Estadual Paulista “Júlio de Mesquita Filho”, Botucatu-SP, Brazil.; II Full Professor, Plastic Surgery Division, Botucatu Medical School, Universidade Estadual Paulista “Júlio de Mesquita Filho”, Botucatu-SP, Brazil.; III PhD, Assistant Professor, Departament of Hematology, Botucatu Medical School, Universidade Estadual Paulista “Júlio de Mesquita Filho”, Botucatu-SP, Brazil.; IV PhD, Assistant Professor, Departament of Pathology, Botucatu Medical School, Universidade Estadual Paulista “Júlio de Mesquita Filho”, Botucatu-SP, Brazil.

**Keywords:** Stem Cells, Microsurgery, Peripheral Nerves, Nerve Regeneration, Rats

## Abstract

**Purpose:**

To evaluate the influence of mesenchymal stem cells from adipose tissue in the end-to-side neurorrhaphy, focusing in the nerve regeneration and the muscle reinnervation in acute trauma.

**Methods:**

140 animals were randomly divided in seven groups: control, denervated, end-to-side neurorrhaphy between distal stump of common peroneal nerve and tibial nerve (ESN), ESN wrapped in fascia, ESN wrapped in fascia and platelet gel, ESN wrapped in platelet gel, ESN wrapped in fascia and platelet gel within stem cells (without culture) removed from the adipose tissue. Mass measurements of the animal and of cranial tibial muscles, electromyography, walking track analysis tests and histological examinations of the nerves and muscles after 180 days was performed.

**Results:**

In the groups where the ESN was performed, the results were always better when compared to the denervated group, showing reinnervation in all ESN groups. The most sensitive methods were walking track and histological analysis. Only the group with stem cells showed values similar to the control group, as well as the functional indices of peroneal nerve and the number of nerve fibers in the peroneal nerve.

**Conclusions:**

Stem cells were effective in ESN according with the functional index of the peroneal nerve, evaluated by walking track analysis and the number of nerve fibers in the peroneal nerve.

## Introduction

Lesions in peripheral nerves are a challenge in the medical practice and cause important repercussions on the socioeconomic sphere^1^–^4^. The best results are achieved through end-to-end suture, which maintains the gold standard treatment for peripheral nerve injuries^3^–^6^. In those cases, in which the proximal stump of the injured nerve is not present, the end-to-side neurorrhaphy is an alternative. The first reports on end-to-side neurorrhaphy are from Ballance^7^, who defended the partial section of the donor nerve, that always led to some functional damage to the territory innervated by the donor nerve. Viterbo*et al*.^8^ proposed the end-to-side neurorrhaphy without donor nerve injury and therefore without functional damage.

Many authors have confirmed neurotropism and the budding or invasion of the distal stump by axons from the donor nerve^8^–^17^. Reinnervation also occurs with antagonistic nerves^18^ and from sensitive nerve to motor nerve^19^, with or without epineurium window^8^^,^ 9 ^,^ 20 ^,^ 21.

In 2017, Viterbo *et al*.^22^ evaluated three different kinds of neurorrhaphy of the peroneal nerve and concluded that in the experimental model used (the neurorrhaphies were made between the stumps of the peroneal nerve), there were no functional or histological differences in the end-to-side, side-to-end and end-to-end techniques of neurorrhaphy.

Even when the reinnervation is proven, the functional results are usually worse than those achieved by the end-to-end neurorrhaphy between stumps of the same nerve, therefore such studies recommend the end-to-side neurorrhaphy for situations where end-to-end neurorrhaphy is not possible or where the proximal stump is not available, as is seen in facial palsy or in global brachial plexus avulsion injuries^6^^,^ 10 ^,^ 13 ^,^ 14.

Stem cells have been used in studies aiming better results in lesions on peripheral nerves, most of these researches have observed the differentiation of stem cells into Schwann cell-like and there are studies using cultured and transplanted Schwann cells for peripheral nerve neurorrhaphy^1^^,^ 2 ^,^ 23–29.

In a recent revision upon the repair of peripheral nerve, Raza *et al*.^3^ considered the vascular aspect of the repair process partially understood but the use of nerve guidance conduits with angiogenic cues still demands the demonstration of its role in functional nerve regeneration. The publication mentioned studies about stem cells in end-to-end neurorrhaphy, others using stem cells from bone marrow and adipose-derived mesenchymal stem cells. No studies about the association of the end-to-side neurorrhaphy with stem cells.

In the search for better results in the end-to-side neurorrhaphy, the aim of this study was to evaluate the influence of mesenchymal stem cells from adipose tissue on nerve regeneration in end-to-side neurorrhaphy.

## Methods

The study was approved by the Animal Ethics Committee of Universidade Estadual Paulista (protocol CEEA 878-2011).

After approved, 140 rats (*Rattus norvegicus*) with eight weeks of life were randomly divided into seven groups (G1 to G7) and operated on.

**Figure 1:**
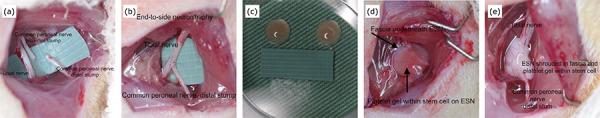
Group 7 (G7) animal experiment. (a) Section of the common peroneal nerve. (b) End-to-side neurorrhaphy. **(c)** Platelet gel containing stem cells. **(d)** Platelet gel containing stem cells on neurorrhaphy, the fascia is below the neurorrhaphy. **(e)** Aspect of the neurorrhaphy surrounded by the fascia.

Group 1(G1): normality control group, the sciatic, tibial and common peroneal right nerves were dissected and identified. Group 2 (G2): control group of denervation, the right common peroneal nerve was sectioned, the proximal and distal stump inverted and fixed in the adjacent musculature. In the groups G3 to G7 the right common peroneal nerve was sectioned, the proximal stump was attached to the surface of the biceps femoralis muscle and the distal stump was sutured to the intact lateral surface of the right tibial nerve, making an end-to-side neurorrhaphy (ESN) with variations in each group. Group 3 (G3): only ESN. Group 4 (G4): a 1 cm^2^ muscle fascia, removed from the right biceps femoris and semitendinosus muscles, wrapped the ESN as an envelope. Group 5 (G5): the same fascia and platelet gel wrapped the ESN. Group 6 (G6):the ESN was wrapped only by platelet gel. Group 7 (G7): theESN was wrapped in fascia and platelet gel that was the carrier of the stem cells obtained from the animal’s own fat (Fig. 1).

The animals were anesthetized with xylazine (30 mg/kg)and ketamine (70 mg/kg) by intramuscular injection. The neurorrhaphy was made with the aid of a microscope DF Vasconcelos (Sao Paulo – SP) under ten augmentations, using four nylon stitches 10.0, always by the same surgeon, the main author. After the procedure, the animals were kept in appropriate cages, receiving water and “ad libitum” feeding, without restrictions of movement and observed for 180 days.

### Obtention of mesenchymal stem cells (CTMs) and platelets gel

Each animal of G7 was anesthetized with xylazine (30 mg/kg) and ketamine (70 mg/kg) by intramuscular injection and 1 g adipose tissue was harvested from the left crural region, immediately packed in a tube containing phosphate buffer solution (PBS - Pierce Biotechnology, Rockford, USA) and sent to Molecular Biology and Cell Engineering Laboratory (UNESP), where the stem cells were identified and prepared to be placed in the neurorrhaphy in G7.

The adipose tissue was rinsed with PBS up to the removal of any remaining blood. The obtained mixture was incubated for 12 hours in a humidified incubator at 37 °C under 5% CO_2_, for the enzymatic digestion in a solution containing 2 mL/g of tissue in a mean of Dulbecco’s modified Eagle medium Knockout (DMEM, Gibco, Grand Island, NY, USA), 2 mg/mL of collagenase type I (Sigma Aldrich, Saint Louis, USA), 20 mg/mL of bovine serum albumin (Invitrogem, Paisey, UK) and 124 mcg of penicillin (Invitrogem, Paisey, UK), followed by neutralization with DMEM containing 10% fetal bovine serum (FBS, Gibico, Grand Islan, NY, USA). This suspension was centrifuged for 5 minutes. The precipitate containing the stromal fraction, rich in mesenchymal stem cells was resuspended in DMEM containing 10% FBS. The mononuclear cells were quantified by hemocytometer to evaluate cellular viability by the Trypan Blue (Sigma Aldrich, Saint Louis, USA) exclusion test. The cells were transported for implantation in a platelet gel in a proportion of 100,000 cells/sample in each G7 animal neurorrhaphy. This mean of cells from the harvested inguinal adipose tissue was found in a previous pilot study sample of 13 animals.

These 13 animals were anesthetized with xylazine(30 mg/kg) and ketamine (70 mg/kg) by intramuscular injection and 1 g of adipose tissue was collected from the left inguinal region (the tissue was processed and the number of stem cells was established in a mean of 100,000). After, the animals were subjected to a intracardiac punch and the blood of the 13 animals was separated in tubes with 3.8% sodium citrate anticoagulant solution in a 1:10 ratio. The first centrifugation was 2400 rpm, the supernatant was collected and subjected to a further centrifugation of 3600 rpm for 15 minutes to obtain the platelet gel that was frozen at –80 °C for use at the time of transfer the stem cells of each animal in group 7.

The cells were microencapsulated in platelets gel in the proportion of 100,000 cells per sample. The clot formed instantly and, thus, a microencapsulated sample was applied to the neurorrhaphy of each animal in G7.

### Cell differentiation and flow cytometry characterized the MSCs

The standard immunohistochemical test was considered positive when the fluorescence was high (≥ 50%), medium (≥ 15 < 50%) or low (≥ 5 < 15%) and negative when the mean fluorescence intensity was very low (< 5%). The positive pattern antibodies were CD71 (FITC mouse anti-rat/BD Pharmigen, San Diego, USA), CD73 (Purified mouse anti-rat/BD Pharmigen, San Diego, USA), CD90 (FITC mouse anti-rat/BD Pharmigen, San Diego, USA), CD105 (PE mouse anti-rat/Life Technology, Carlsbad, CA) and CD106 (Purified mouse anti-rat/BD Pharmigen, San Diego, USA). The negative pattern antibodies were CD31 (mouse PE anti-rat/BD Pharmigen, San Diego, USA), CD34 (rabbit FITC anti-rat/Biorbyt, Saint Louis, USA), CD40 (FITC hamster anti-rat/BD Pharmigen, Saint Louis, USA), CD44 (RPE mouse anti-rat/AbD Serotec, Kidlington, UK), CD45 (FITC mouse anti-rat/BD Pharmigen, Saint Louis, USA) and CD11b (Biotin mouse anti-rat/BD Pharmigen, Saint Luis, USA).

A random sample of mesenchymal stem cells was cultured for differentiation in the adipogenic, osteogenic and chondrogenic lines. This culture was performed only to confirm the ability of the cells to differentiate but not to be used in the experiment. The culture was performed according to a pre-established protocol in the Molecular Biology and Cell Engineering Laboratory for mesenchymal stem cells obtained from the adipose tissue. Evaluations were performed in a blind way.

### Mass of the animals and cranial tibial muscles

The body mass of each animal was measured before and after the experiment.

The cranial tibial muscle, innervated only by the common peroneal nerve, was harvested from the left (normal) and the right sides (experimental). The masses of the right and the left cranial tibial muscles were measured at the end of the experiment.

### Walking track analysis

The gait evaluation test by walking track was performed every 30 days. The animals, previously trained, had their hind paws painted with India ink and walked down on a white sheet of paper placed on the floor of a corridor of 9 by 78 cm. The footprints marked on the paper were analyzed by print length (PL) and toe spread (TS) of the experimental (right side) and normal (left side) hind paws. These measures were used in the calculation of the functional index of the peroneal nerve (PFI) according to Bain *et al*.^30^. The used formula was: PFI = 174.9 × (EPL – NPL)/NPL + 80.3 × (ETS – NTS)/NTS – 13.4; NPL is normal print length (left side), EPL is experimental print length (right side), NTS is normal toe spread (left side) and ETS is experimental toe spread (right side).

### Electrophysiological test

At 180 days, after anesthetized, the electrophysiological test of the right cranial tibial muscles was performed using the Sapphire II 4ME electromyograph (Medelec/TECA - USA). The active electrode was placed in the cranial tibial muscle and the reference electrode in the tendon of this muscle; a dispersive electrode was placed in the trunk. The stimulus had a frequency of 1 pps (pulse per second), with the duration of 100 μs and intensity of 5.1 V, applied by a bipolar electrode, which cathode and anode were 2 mm apart, positioned on the sciatic nerve before the neurorrhaphy. The amplitude and latency of muscle action potential were measured. The electrophysiological test was performed with the tibial and sural nerves intact. After that, both nerves were sectioned to make sure that the stimulus that arrived to the cranial tibial muscles was passing only through the neurorrhaphy between the common peroneal and the tibial nerves. This procedure was described by Viterbo *et al*. 6–8. The amplitude measure of greatest value and its corresponding latency among six measures was chosen in each animal. These values measured after the section of sural and tibial nerves were considered to the comparison among groups.

### Histological analysis

After the electrophysiological test and a lethal dose of pentobarbital sodium, nerves and muscles were harvested.

The nerve segments were identified as N1, N2 and N3 and the muscles by M. Nerve segment 1 (N1): distal stump of the common peroneal nerve after neurorrhaphy, N2: proximal stump of the common peroneal nerve and N3: area of neurorrhaphy. Muscles (M): right and left cranial tibial muscles. In G1, N1 and M were collected. In G2, samples of N1, N2 and M were collected. From G3 to G7, N1, N2, N3 and M were collected.

The cranial tibial muscles were fixed in liquid nitrogen and submitted to cross sections of 7 μm in cryostat (Leica CM 1850). The sections were stained by hematoxylin-eosin technique (HE). These images were analyzed using the Scanscope scanner (2003, Leica Biosystems V11.2.0.780). Five fields were chosen under 10 magnification, one in each corner and one at central field. The muscle fibers were analyzed for measurements of area, perimeter and minimum diameter.

The images from the nerve specimens’ sections were analyzed using the Pannoramic Viewer (2012, 3DHISTECH Ltda. V1.15.3).

The specimens of N1 and N2 were fixed in Karnovisk and prepared in historesins. The N2 nerve specimens were submitted to longitudinal sections and stained with the Bielschowsky technique in order to observe the occurrence of neuroma of amputation.

The N1 nerve specimens were cross-sectioned and stained with 0.25% toluidine blue. The number of nerve fibers was counted in all sections. The nerve fibers area and the minimum diameter were evaluated. Five fields were analyzed on each section, one in each corner and one in the central field in × 40 magnification.

The N3 segments were fixed in 10% buffered formol, paraffin embedded and stained by Hematoxylin-Eosin. The presence of vessels, hemorrhage, edema and inflammatory reaction was searched. A quantitative morphological evaluation from 0 to 4 crosses was performed: zero (0) was the absence of the evaluated elements, one (1) discrete quantity, two (2) moderate amount, three (3) large quantity and (4) intense quantity of the evaluated elements. The type of cells present in the inflammatory reaction was evaluated.

Also, in N3, the antibodies used in the immuno-histochemical study were: CD105 (ORB10285 – Biorbyt, Saint Louis, USA), used to evaluate the formation of new vessels; CD34 (ORB27549 – Biorbyt, Saint Louis, USA), to evaluate the existing vascularization; the protein S100 (rabbit Polyclonal, Novus Biologicals, Centennial, USA), to evaluate the presence of Schwann cells and CD90 (HIS51 - Novus Biologicals, Centennial, USA), to identify mesenchymal stem cells. For the reading of the immunohistochemical study a score of the reaction of the markers was used. The intensity of the variation was considered from 0 to 3: zero (0) for no reaction, one (1) for low intensity, two (2) for moderate intensity and three (3) for strong intensity. As to the extension, the variation was from 0 to 3: zero (0) was considered when there was no reaction, one (1) when the extension was up to one third of the section, two (2) when the extension was from one to two thirds of the section and three (3) if the extension of the reaction was seen above two thirds.

### Statistical analysis

For most statistical analysis, groups were compared by the analysis of variance ANOVA and Tukey’s test. For the analysis of the number of cells in N1 a generalized linear model with negative binomial distribution and Wald multiple comparison test were used. In the segment of neurorrhaphy nerves (N3) in which edema, hemorrhage, vessels and inflammation were analyzed, and for the analysis by immunohistochemistry, the nonparametric test of Median was used. A p < 0.05 was considered significant for all analysis.

## Results

Table 1 shows the result of the characterization of the stem cells by flow cytometry. Samples of stem cells harvested from the adipose tissue of 13 animals in the pilot study and 20 animals in group 7 (33 samples total) were characterized by flow cytometry to determine their phenotype. An average of 10,677 events (cells) were analyzed, within the standard of excellence for data interpretation. The mean intensity of negative fluorescence was 0.93% (negative control). Negative (which must not be expressed in stem cells) and positive (which must be expressed in stem cells) markers were used. The CD11b, CD31, CD34 and CD40 markers showed the expected result, with medium or low immunofluorescence average, as well as the CD90, CD105 and CD106 markers, with medium or high immunofluorescence average, allowing the characterization of stem cells through their phenotypic profile. The culture for differentiation in the adipogenic, osteogenic and chondrogenic lines was confirmed.

**Table 1:** Percentage evaluation of the expression of the markers used in flow cytometry in the samples of the animals of group 7.

Marker expression level				High		Medium		Low		Negative
Pattern		MIF (%)		≥ 50		≥ 15 and < 50		≥ 5 and < 15		< 5
Negative pattern		Control		0		0		0		33 (100%)
		CD11b		2 (6%)		26 (78.7%)		5 (15.15%)		0
		CD31		1 (3%)		17 (51.5%)		14 (42.42%)		1 (3%)
		CD34		0		0		2 (6.06%)		31 (93.93%)
		CD40		0		0		0		33 (100%)
		CD44		17 (51.51%)		16 (46.48%)		0		0
		CD45		1 (3.03%)		28 (84.8%)		2 (6.06%)		2 (6.06%)
										
Positive pattern		CD71		0		1 (3.0%)		24 (72.72%)		8 (24.24%)
		CD73		0		19 (57.57%)		14 (42.42%)		0
		CD90		13 (39.39%)		20 (60.60%)		0		0
		CD105		12 (36.36%)		21 (63.63%)		0		0
		CD106		8 (24.24%)		24 (72.72%)		1 (3%)		0

MIF: % of the mean immunofluorescence.

There was no difference between the groups regarding the initial or final body mass of the animals. As for the right cranial tibial muscle mass, there was no difference between the G3 and G7 groups, but these groups were worse than the control group (G1) and better than the denervated group (G2). As for the mass of the left cranial tibial muscle, there was no difference between groups G1 to G7. In each group the right cranial tibial muscle presented a lower mass than the left cranial tibial muscle in the G2 to G7 groups (Table 2), p < 0.05.

**Table 2:** Mass of the animals before and after the experiment, mass of the cranial tibial muscles, right and left, electrophysiological test and walking track analysis at 180 days (peroneal functional index).

Groups	Mass of animals (g)				Mass of cranial tibial muscles (g)							Electrophysiological Test						Walking Track Analysis		
		Before	After				Right			Left				Latency	Amplitude					180 days
G1		276.95 ±28.37	461.00 ±50.21				0.94 ± 0.11			0.90 ± 0.15				2.02 ± 0.54	9.66 ± 4.94					-15.21 ± 20.71
		A	B				A		a	A		a		C	A					A
G2		265.28 ±34.00	464.80 ±66.25				0.24 ± 0.19			0.89 ± 0.16				5.62 ± 3.14	0.91 ± 1.52					-154.86 ±17.41
		A	B				C		a	A		b		A	C					D
G3		279.90 ±29.52	467.27 ±72.99				0.55 ± 0.19			0.87 ± 0.16				2.34 ± 1.34	8.93 ± 5.63					-75.16 ± 51.78
		A	B				B		a	A		b		B	AB					C
G4		271.05 ±26.14 A	490.18 ±51.62 B				0.64 ± 0.18			0.89 ± 0.07				1.96 ± 0.37	8.86 ± 3.36					-82.85 ± 52.0
		A	B				B		a	A		b		C	AB					C
G5		279.25 ±19.47	492.45 ±43.27				0.58 ± 0.12			0.89 ± 0.09				2.60 ± 1.05	7.37 ± 4.41					-70.83 ± 60.28
		A	B				B		a	A		b		B	AB					BC
G6		273.95 ±23.48	483.32 ±53.33				0.62 ± 0.17			0.87 ± 0.12				2.66 ± 1.03	6.99 ± 2.93					-66.18 ± 60.5
		A	B				B		a	A		b		B	AB					BC
G7		271.3 ±17.98	495.55 ±38.94				0.68 ±0.10			0.88 ±0.06				2.30 ± 1.02	5.67 ± 2.89					-31.48 ± 24.68
		A	B				B		a	A		b		BC	B					AB

G1 (control group), G2 (denervated), G3 (ESN), G4 (ESN and fascia), G5 (ESN, fascia and platelet gel), G6 (ESN and platelet gel), G7 (ESN, fascia, platelet gel and stem cells). This table shows mean and standard deviation (e.g., 276.95 ± 28.37) followed by an uppercase letter that compares the groups among themselves in the same column for each parameter. The mass of the animals before the experiment: the first column, the mass of the animals after the experiment: the second column, the mass of the cranial tibial muscle removed from the right and from the left paw: the third and the fourth columns, the electrophysiological test: columns fifth and sixth and for the walking track analysis: the eighth column. Uppercase letters show the comparison among the groups in each column, different uppercase letters show statistical difference in each parameter, p < 0.05. Lowercase letters compare the mass of the cranial tibial muscles between the right and left sides within the same group, different lowercase letters show statistical difference, p < 0.05. Analysis of variance and Tukey’s test were used for the analysis.

In the evaluation of the latency (electrophysiological test), the groups G1, G4 and G7 presented the best results, p < 0.05. For amplitude, G3 to G7 did not differ statistically from each other, G2 and G7 presented lower results than G1 (Table 2), p < 0.05.

**Figure 2:**
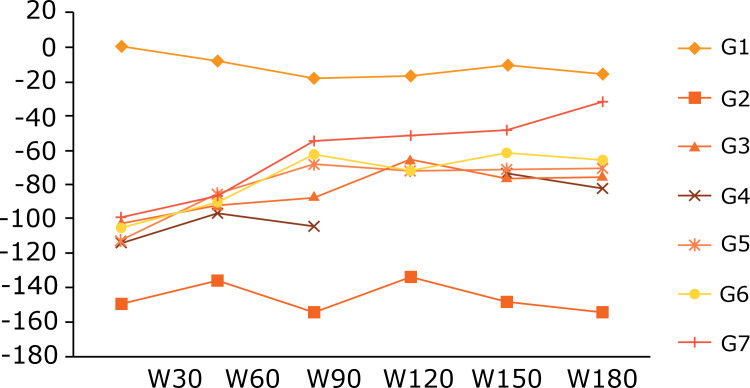
Graphic of the functional index of the peroneal nerve at 30 days intervals according to the gait evaluation test walking track analysis. W = Walking track analysis, W30 = 30 days, W60 = 60 days, W90 = 90 days,W120 = 120 days, W150 = 150 days and W180 = 180 days.G1 (control group), G2 (denervated), G3 (ESN), G4 (ESN and fascia), G5 (ESN, fascia and platelet gel), G6 (ESN and platelet gel), G7 (ESN, fascia, platelet gel and stem cells).

In the walking track analysis, the results of peroneal functional index (PFI) at 120 to 180 days, only G7 (ESN wrapped in fascia and platelet gel within stem cells) presented values statistically similar to G1 (Table 2, Fig. 2), p < 0.05. In group 4, data collected at 120 days are missing, so there is a gap in the graph of Fig. 2.

In the study of morphology and morphometry of the cranial tibial muscles, the muscle fibers of the control group (G1) presented a polygonal shape, nucleus in a peripheral position and little connective tissue among the fibers. In G2, more connective tissue among the muscle fibers which were smaller was observed and the cellular infiltrate was abundant. Regarding the minimum diameter and area of the muscle fibers on the right, groups G3 to G7 showed results superior to G2 and inferior to G1. Regarding the perimeter of the right cranial tibial muscle, the worst result was of G2 and the best results were of G4, G5, G7 and G1. Only groups G1, G6 and G7 did not show statistical difference between the right and left sides for minimum diameter, area and perimeter (Table 3), p < 0.05.

**Table 3:** Histomorphological assessments in right and left cranial tibial muscles and common peroneal nerve (N1), distal to neurorrhaphy.

Right and left cranial tibial muscles													Common Peroneal nerve					
Groups	Minimum diameter (μm)				Perimeter (μm)				Area (μm^2^)					Number of fibers		Area (μm^2^)		Minimum diameter (μm)
		R		L		R		L		R		L						
G1		46.91 ± 3.96 Aa		47.83 ± 7.34 Aa		234.68 ± 20.01 Aa		239.26 ± 29.47 Aa		3275.48 ± 504.38 Aa		3346.61 ± 707.77 Aa		1909.67 ±525.87 A		72.49 ±15.79 A		7.48 ± 1.09 A
G2		23.44 ± 4.79 C a		46.09 ± 3.94 ABb		117.24 ± 28.12 Ca		224.01 ± 25.86 ABb		870.48 ± 448.67 Ca		3108.06 ± 613.4 ABb						
	G3		41.32 ± 5.71 Ba		45.91 ± 4.55 ABb		204.59 ± 29.41 Ba		223.91 ± 28.22 ABb		2547.94 ± 620.72 Ba		3077.27 ± 741.65 ABb		1154.56 ± 864.49 BC		32.98 ± 6.25 C		4.96 ± 0.35 C
G4		42.01 ± 4.44 Ba		46.82 ± 4.04 ABb		217.56 ± 26.27 ABa		241.89 ± 22.48 Ab		2702.79 ± 547.71 Ba		3413.74 ± 560.98 Ab		1015.61 ± 782.03 BC		30.25 ± 9.54 C		4.49 ± 0.75 D
G5		41.57 ± 4.47 Ba		46.32 ± 5.6 ABb		212.69 ± 23.04 ABa		235.64 ± 31.59 ABb		2634.43 ± 503.67 Ba		3284.25 ± 771.74 ABb		905.20 ± 609.25 C		31.85 ± 6.39 C		4.89 ± 0.55 C
G6		41.4 ± 5.26 Ba		43.16 ± 3.18 Ba		205.81 ± 28.82 Ba		212.36 ± 12.87 ABa		2559.75 ± 583.6 Ba		2718.2 ± 324.4 Ba		1210.5 ±1364.18 BC		39.08 ±13.59 B		5.44 ± 0.86 B
G7		42.15 ± 3.42 Ba		43.93 ± 3.93 ABa		214.06 ± 18.05 ABa		225.11 ± 22.6 ABa		2675.17 ± 462.67 Ba		2973.3 ± 555.58 ABa		1437.44 ± 701.73 AB		30.36 ± 9.45 C		4.75 ± 0.81 CD

G1 (control group), G2 (denervated), G3 (ESN), G4 (ESN and fascia), G5 (ESN, fascia and platelet gel), G6 (ESN and platelet gel), G7 (ESN, fascia, platelet gel and stem cells). R: right, L: left. This table shows mean and standard deviation (e.g., 46.91 ± 3.96) followed by an uppercase letter that compares the groups among themselves in the same column. The lowercase letters compare the sides right and left in the same group, for each parameter, in the same row. Different letters show statistical difference. Analysis of variance and Tukey’s test were used for the analysis of the minimum diameter, the perimeter and area of muscles and for the area and minimum diameter of common peroneal nerve, p < 0.05. For the analysis of the number of fibers in common peroneal nerve, a generalized linear model with negative binomial distribution and Wald multiple comparison test were used, p < 0.05. In G2, the common peroneal nerve, distal to neurorrhaphy, was not analyzed because it was too degenerated.

**Figure 3:**
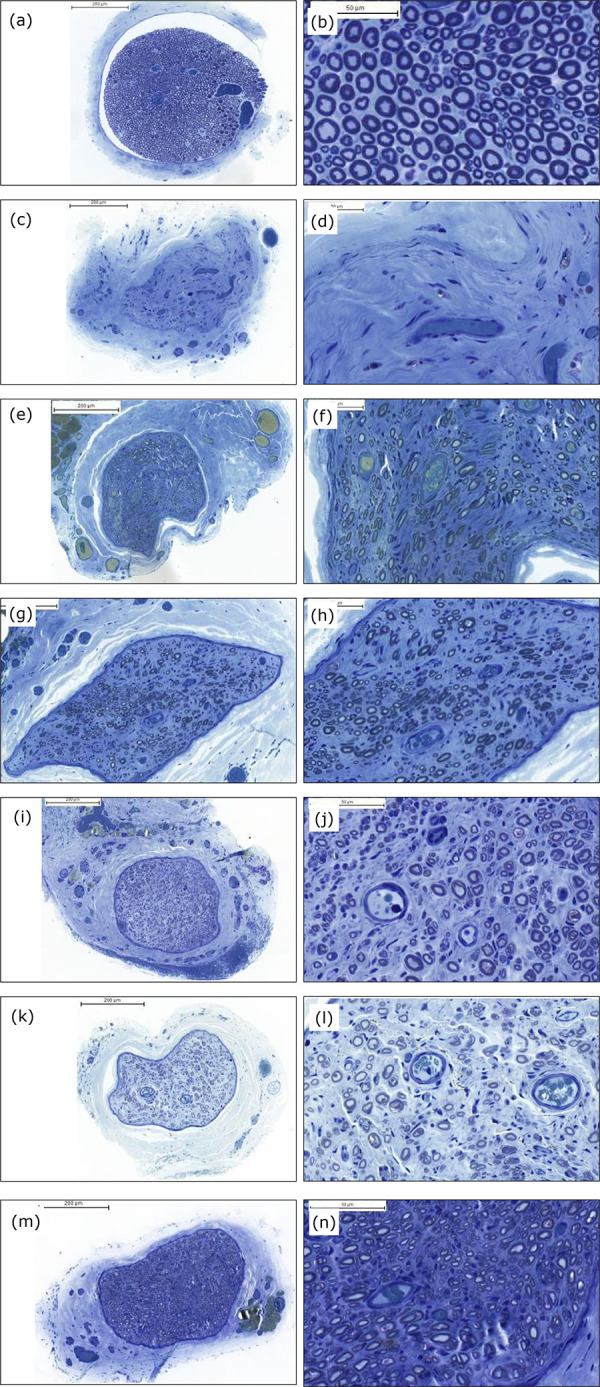
(a), (c), (e), (g), (i), (k) and (m) right common peroneal nerves (N1) after neurorrhaphy (magnification × 10). (b), (d), (f), (h), (j), (l) and (n) right common peroneal nerves after neurorrhaphy (N1), (magnification × 40). Toluidine blue. (a) and (b): G1, control group; (c) and (d): G2, denervated; (e) and (f): G3, ESN; (g) and (h): G4, NTL and fascia; (i) and (j): G5, NTL, fascia and platelet gel; (k) and (l): G6, NTL and platelet gel; and (m) and (n): G7, NTL, fascia, platelet gel and stem cell.

As to the number of nerve fibers observed in N1 segment (distal stump of the common peroneal nerve after neurorrhaphy), only G7 presented a result statistically similar to G1, although it did not differ from G3, G4 and G6 groups. As for the area of the nerve fiber and the minimum diameter of the nerve fiber, G6 presented the best result among groups G3 to G7, but worse than G1 (Table 3, Fig. 3), p < 0.05.

In proximal stumps of the common peroneal nerve (N2), amputation neuroma formation was observed in all samples from G2 to G7.

Analyzing the neurorrhaphy (N3) for hemorrhage, edema and inflammation, the G3 to G7 groups did not differ statistically from each other. As for the vessels observed in this same segment, G7 presented a statistically similar result to G3 and better than the results of groups G4, G5 and G6 that did not differ from each other and from G3 (Table 4), p < 0.05.

**Table 4:** Histological evaluations of the G3 to G7 groups by immunohistochemistry evaluated the median of a score varying from 0 to 3 for the intensity and extent of S100, CD90, CD34 and DC105 antibodies in neurorrhaphy (N3) and common peroneal nerve. Hemorrhage, edema, inflammation and presence of vessels in the N3 segment stained with hematoxylin and eosin compared to the median of a score ranging from 0 to 4 in the G3 to G7 groups.

	Groups							G3		G4		G5		G6		G7
Immunohistochemistry		Perineurorrhaphy		S100		Intensity		2 AB		2 AB		2 AB		2 B		3 A
						Extension		2 AB		2 AB		2 AB		2 B		3 A
				CD 90		Intensity		0 B		0 B		0 AB		0 B		2 A
						Extension		0 B		0 B		0 AB		0 B		2 A
				CD 34		Intensity		2 A		1 A		2 A		2 A		2 A
						Extension		3 A		2 A		2 A		2 A		3 A
				CD105		Intensity		3 A		1 A		2 A		2 A		3 A
						Extension		3 A		1 A		2 A		2 A		3 A
															
		Common Peroneal nerve		CD34		Intensity		0.5 B		2 AB		2 AB		1 B		3 A
						Extension		0.5 B		2 AB		1.5AB		1 B		3 A
				CD105		Intensity		0.5 B		2 AB		1.5AB		1 B		3 A
						Extension		0.5 B		2 AB		1 AB		1 B		3 A
																
Hematoxylin and eosin		Perineurorrhaphy				Bleeding		1 A		1 A		2 A		2 A		1 A
						Edema		1 A		1 A		1 A		1 A		1 A
						Inflammation		2 A		1 A		1 A		1 A		0 A
						Vases		2 AB		2 B		2 B		2 B		4 A

G1 (control group), G2 (denervated), G3 (ESN), G4 (ESN and fascia), G5 (ESN, fascia and platelet gel), G6 (ESN and platelet gel), G7 (ESN, fascia, platelet gel and stem cells). The comparison was made by the nonparametric median, p < 0.05. Different letters show statistical difference.

**Figure 4:**
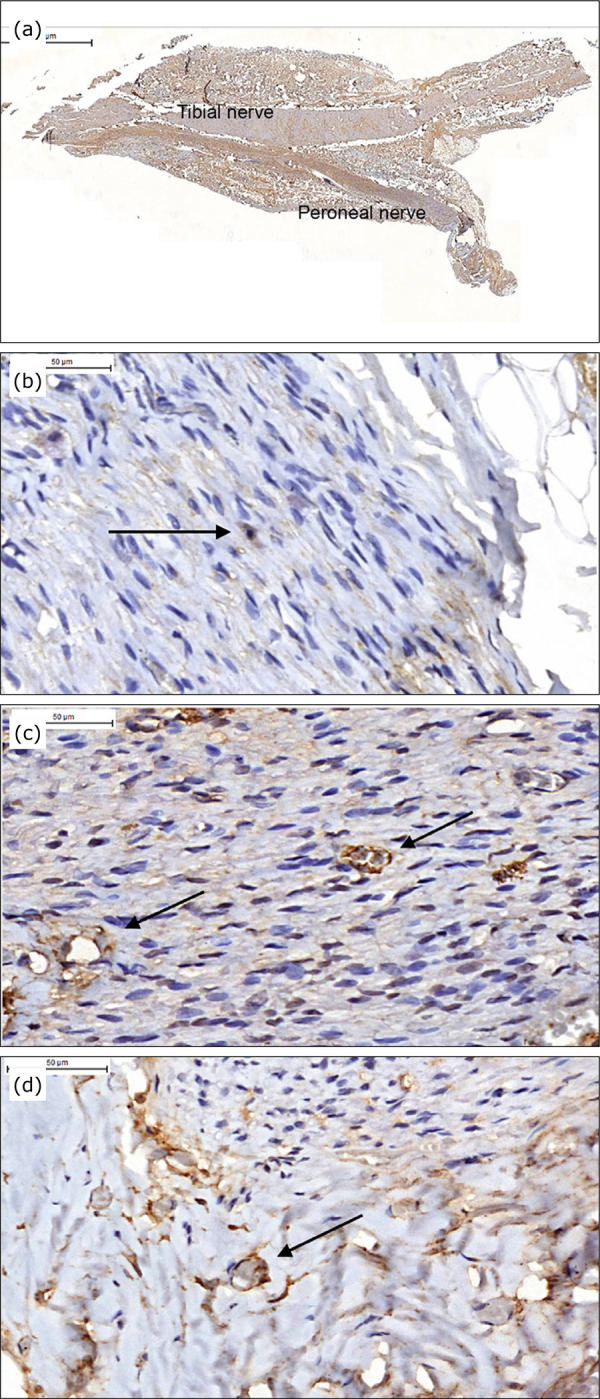
Animal of G7 (NTL, fascia, platelet gel and stem cell). Images of immunohistochemical (IMH) study. (a) Segment of the neurorrhaphy marked by the S100, showing greater intensity in the common peroneal nerve. (b) CD90-labeled mesenchymal stem cell. (c) Vessels marked by CD105 within the common peroneal nerve. (d) Vessels marked by CD34 perineurorrhaphy. A (IMH-S100/× 1), B (IMH-CD90/× 20), C (IMH-CD105/× 40), D (IMH-CD34/× 40).

Regarding the intensity and extent of the S100 protein in N3, G7 was statistically superior than G6, not statistically differing from the other groups, which did not differ from each other. Regarding the CD90 marker in N3, the best results were seen in G5 and G7. As to the intensity and extent of the CD34 and 105 markers analyzed in the N3 perineurorrhaphy region, there was no statistical difference between G3 and G7. As to the intensity and extent of the CD34 and 105 markers analyzed in the fibular nerve segment in N3, there was no superiority of any group (Table 4, Fig. 4), p < 0.05.

## Discussion

Mesenchymal stem cells from adipose tissue have the potential for transdifferentiation in the Schwann cell or potential to aid in peripheral nerve regeneration by other mechanisms^2^^,^ 23 ^,^ 31–35 and are available in large quantities. The number of stem cells to be used in a peripheral nerve injury experiment is not established. In the literature, there are studies that implanted from 4 × 10^3^ to 2 × 10^7^ cells^2^^,^ 24 ^,^ 25 ^,^ 27 ^,^ 36–38. In this model were used 1 × 10^5^ cells that were not cultured, thinking about the possibility of using them in an acute trauma condition. The number of cells (1 × 10^5^) was established after a pilot study in a group of 13 animals, where the average number of mesenchymal stem cells found was 1 × 10^5^ cells in 1 g of fat tissue harvested from the inguinal region.

The criteria used to confirm the presence of mesenchymal stem cells among the transferred cells were: plastic adherence capacity, flow cytometric immunophenotyping and differentiation of three strains^39^^,^ 40.

CD34 and CD40 antibodies were clearly negative for these cells. CD11b and CD31 showed fluorescence around 25%, due to the fact that samples do not present purging under the cell culture method. CD 44 and CD 45 presented medium and high fluorescence, as the stem cells did not come from a culture, did not undergo purging, presenting high reaction to the leukocytes and polymorphonuclear of the sample. CD90, CD105 and CD106 were clearly positive for the sample. CD71 and CD73 presented medium or low fluorescence, but positive.

Platelet gel was used as a suitable mean for the transfer and deposition of stem cells in the area of neurorrhaphy. The G5 (ESN, platelet gel and fascia) or G6 (ESN and platelet gel) groups did not show better results than the others, G3 or G4, so the platelet gel does not seem to positively influence the reinnervation or peripheral nerve regeneration in this experimental model. Similar results were suggested by Braga-Silva *et al*.^26^ in another study in rats.

The fascia was used involving the neurorrhaphy in order to ensure that the gel containing the stem cells would be maintained around the neurorrhaphy.

Similar studies in end-to-side neurorrhaphy adopted periods around 12 weeks of observation for functional tests^10^^,^ 14 ^,^ 41 ^,^ 42. The observation time of around 24 weeks used in this study was more appropriate, since it allowed the observation of a progressive improvement in the functional index of the peroneal nerve, evaluated by walking track analysis from 17 to 24 weeks (from 120 to 180 days). It became clear that the nerve regeneration and the muscular reinnervation was obtained after 12 weeks.

The animals were comparable in weight at the beginning and at the end of the experiment, excluding differences in the result from unequal mass gain. Regarding the mass of the cranial tibial muscles, on the right side, the results showed that there was reinnervation that allowed recovery of muscle mass in all groups where ESN was performed. All groups showed recovery on the side where the neurorrhaphy was performed (right), with values significantly higher than the denervated group (G2), although none of them resembled G1 (normality control group), result similar to those found in other studies^8^–^10^^,^ 12 ^,^ 19 ^,^ 42. It is important to remember that the cranial tibial muscle is innervated exclusively by the common peroneal nerve, so the recovery of muscle trophism depended exclusively on end-to-side neurorrhaphy^8^.

For the electrophysiological test it was established that the section of tibial and sural nerves should be made, in order to avoid passage of nerve stimulation by possible neurotrophism with the cranial tibial muscle during the experiment. An intriguing result was found: as for amplitude, only group 7 presented a lower result than the control group (but significantly higher than the denervated group, G2); as for latency, group 4 presented a result significantly similar to group 1, as well as group 7, that did not differ from the other groups (G3, G5 and G6). This was considered an isolated result. Based on the results, the electrophysiological test was not sensitive in this study. Viterbo *et al*.^22^, repeating a similar methodology in the same experimental model of this study, also failed to differentiate the experimental groups from each other, only confirming the reinnervation in the experimental groups.

Functional tests, used since Medinaceli^43^, are considered excellent in the evaluation for it demonstrates the evolution in motor recovery^44^, the ability of nerve and muscular regeneration in a functional way^22^^,^ 45, besides the muscular readaptation and cerebral neuroplasticity, when antagonistic nerves are used in the reinnervation^18^.

Functional tests are cheap and easily reproducible^44^. The footprints can be analyzed (measured) and revised or checked at any time and the possibility of being performed on several occasions during the experiment makes it possible to assess the progression of reinnervation. In a study on functional assay, Nichols *et al*.^45^ stated that although peripheral nerve reinnervation can be assessed from electrophysiology and histomorphometry, the benchmark of successful reinnervation remains the functional recovery and also that recovery of function does not necessarily correspond to histologic and electrophysiologic evidence of regeneration.

For the functional index of the peroneal nerve from 120 to 180 days, only G7 showed a statistically comparable result to the G1 result (normality control group).

Motor recovery was seen during the course of the study (Fig. 2) and confirmed statistically at the end of it with the statistical similarity of group 7 to the control group at 180 days. According to Nichols *et al*.^45^, this is the most important result in this study.

The minimum diameter of the muscular fibers is the most reliable measure for the comparison of the groups in the analysis of the muscular fibers, since this parameter does not suffer interference of the inclination of the transversal cut of the muscular fibers^46^. For the minimum diameter, perimeter and area of the muscle fibers on the right side, the G3 to G7 groups showed no difference among them, with better results than that observed in the denervated group. Therefore, there was reinnervation and maintenance of muscular trophism, a similar result to those found in other studies^9^^,^ 20 ^,^ 42. For the same parameters, minimum diameter, perimeter and area, when comparing the right and left sides in the same group, groups G6 and G7 showed similar behavior to G1 (normality control group), with no statistical difference between the sides, the best results were found in G1, G6 and G7, p < 0.05.

As for the number of nerve fibers in the N1 segment, only G7 statistically resembled G1, although G7 was not statistically different from G3, G4 and G6 groups. In similar studies on ESN, the number of nerve fibers in the group where the end-to-side neurorrhaphy was performed is statistically lower than that found in the control group^8^^,^ 9 ^,^ 16 ^,^ 19 ^,^ 20 ^,^ 42. Studies with stem cells in peripheral nerve regeneration have shown the tendency to increase nerve fibers in the group treated with stem cells^36^^,^ 47, a similar result to that found in this study. So, this result refers to the possible influence of stem cells on the number of axons, this result is considered to be important, since it is about counting the total number of fibers and not just the sample of each animal.

There was no major inflammatory reaction in the group where stem cells were used (G7), considering the results of the histological parameters studied in N3 (Table 4).

The S100 protein antibody evaluates the presence of Schwann cells. The site of observation was intra nerves and on the periphery of neurorrhaphy. The G7 group showed no superior results than the ones from groups G3 to G6, although many studies have observed the differentiation of stem cells into Schwann cells or Schwann cell-like^1^^,^ 2 ^,^ 23–29 ^,^ 47. This may be that the greatest importance of mesenchymal stem cells is the production of neurotrophic factors and not their transdifferentiation in Schwann cells, as suggested by Cartarozzi *et al*.^48^ as well as by this study, because there was no difference among the groups regarding immunohistochemical analysis by the S100 marker.

The CD90 marker identifies mesenchymal stem cells. The best results were observed in groups G5 and G7. Perhaps the initial effect would have been important and the result of the analysis of these parameters would have been different if some of these animals had been sacrificed in the first weeks of the experiment. These results correspond to the results found by Erba *et al*.^32^, who affirmed that the number of mesenchymal stem cells derived from fat had decreased significantly in number after 14 days of implantation in sciatic nerve injury in rats.

The CD105 marker identifies neovascularization and CD34 evaluates the previously existing vascularization in each sample. Increased vascularization was observed in all groups from G3 to G7, so there was no association between the use of platelet gel or stem cells and the increased vascularization.

Other studies using stem cells removed from bone marrow in different experimental models of peripheral nerve injury observed better results in the group that used stem cells compared to the other groups to improve gait function^26^^,^ 27, greater number of regenerated axons or myelinated fibers^26^^,^ 27 ^,^ 36, faster regenerative process^26^^,^ 36 ^,^ 38, less muscle loss^27^, greater myelin area^36^ and less Wallerian degeneration^38^. In a meta-analysis study on the use of stem cells in the regeneration of peripheral nerve defects, the regenerative effect of stem cells was demonstrated mainly by analysis of the mass of the muscles studied, electrophysiological tests and gait tests^34^.

In the present study, it was clear that the end-to-side neurorrhaphy was effective as a form to repair the peripheral nerve injury. Although having been used in a number lower than that possibly obtained by a culture, the mesenchymal stem cells obtained from the adipose tissue points to better results by presenting values similar to those found in the control group, regarding the functional index of the peroneal nerve and number of nerve fibers.

These results indicate that the use of allogeneic platelet gel and fascia can effectively serve as a scaffold for mesenchymal stem cells in studies on peripheral nerve regeneration. The use of fascia and allogeneic platelet gel were relevant factors for the transfer and maintenance of the stem cells at the site of the neurorrhaphy but did not influence the result.

This is the first study done with mesenchymal stem cells derived from adipose tissue and fascia in end-to-side neurorrhaphy. Other similar studies are necessary to confirm these results. With other promising results, stem cells derived from adipose tissue may have their clinical application in acute lesions of peripheral nerves.

## Conclusion

The group in which stem cells obtained from the adipose tissue were used presented a functional index of the peroneal nerve, evaluated by walking track analysis and the number of nerve fibers in the peroneal nerve, similar to the control group of normality.
